# Supplementary material for: A Critical View of the Application of the APEX Software (Aqueous Photochemistry of Environmentally-Occurring Xenobiotics) to Predict Photoreaction Kinetics in Surface Freshwaters
Source: Molecules. 2019 Dec 18;25(1):9. doi: 10.3390/molecules25010009 (PMC7017383; doi:10.3390/molecules25010009)
Supplement: Supplementary file 1 [file molecules-25-00009-s001.zip › 3.2.4_gcc-4.4.0.zip!/3.2.4_gcc-4.4.0/3.2.4_gcc-4.4.0/doc/octave/html/interpreter/Customizing-the-Prompt.html]

Customizing the Prompt - Untitled


Next: Diary and Echo Commands,
Previous: Customizing readline,
Up: Command Line Editing


---

#### 2.4.7 Customizing the Prompt

The following variables are available for customizing the appearance of
the command-line prompts. Octave allows the prompt to be customized by
inserting a number of backslash-escaped special characters that are
decoded as follows:

\t: The time. \d: The date. \n: Begins a new line by printing the equivalent of a carriage return followed by a line feed. \s: The name of the program (usually just octave). \w: The current working directory. \W: The basename of the current working directory. \u: The username of the current user. \h: The hostname, up to the first `.'. \H: The hostname. \#: The command number of this command, counting from when Octave starts. \!: The history number of this command. This differs from \# by the number of commands in the history list when Octave starts. \$: If the effective UID is 0, a #, otherwise a $. \nnn: The character whose character code in octal is nnn. \\: A backslash.

— Built-in Function: val = **PS1** ()  
— Built-in Function: old\_val = **PS1** (new\_val)  
> Query or set the primary prompt string. When executing interactively,
> Octave displays the primary prompt when it is ready to read a command.
>
> The default value of the primary prompt string is `"\s:\#> "`.
> To change it, use a command like
>
> ```
>           octave:13> PS1 ("\\u@\\H> ")
> ```
>
> which will result in the prompt boris@kremvax>  for the user
> boris logged in on the host kremvax.kgb.su. Note that two
> backslashes are required to enter a backslash into a double-quoted
> character string.
> See Strings.
>
>
>
> **See also:** PS2, PS4.

— Built-in Function: val = **PS2** ()  
— Built-in Function: old\_val = **PS2** (new\_val)  
> Query or set the secondary prompt string. The secondary prompt is
> printed when Octave is expecting additional input to complete a
> command. For example, if you are typing a `for` loop that spans several
> lines, Octave will print the secondary prompt at the beginning of
> each line after the first. The default value of the secondary prompt
> string is `"> "`.
>
>
>
> **See also:** PS1, PS4.

— Built-in Function: val = **PS4** ()  
— Built-in Function: old\_val = **PS4** (new\_val)  
> Query or set the character string used to prefix output produced
> when echoing commands is enabled.
> The default value is `"+ "`.
> See Diary and Echo Commands, for a description of echoing commands.
>
>
>
> **See also:** echo, echo\_executing\_commands, PS1, PS2.
